# Relationship between albumin and osteoporosis in patients with type 2 diabetes mellitus

**DOI:** 10.3389/fendo.2025.1449557

**Published:** 2025-04-14

**Authors:** Hang Zhao, Cuijuan Qi, Yunjia Zhang, Liqun Yang, Luping Ren, Shuchun Chen

**Affiliations:** Department of Endocrinology, Hebei General Hospital, Shijiazhuang, Hebei, China

**Keywords:** albumin, osteoporosis, bone mineral density, type 2 diabetes mellitus albumin, type 2 diabetes mellitus

## Abstract

**Purpose:**

This study aimed to investigate whether low albumin levels are a risk factor for osteoporosis (OP) in patients with type 2 diabetes mellitus (T2DM).

**Patients and methods:**

This study included adult patients with T2DM hospitalized at the Hebei General Hospital in China between 2019 and 2020. Patients were divided into groups based on OP to explore the differences between the two groups and stratified by sex. Confounding factors were screened based on the statistical results and clinical practice, and regression analysis was used to explore the relationship between albumin levels and OP.

**Results:**

In total, 125 patients (68 men and 57 women) with T2DM were included in this study. Triglyceride and high-density lipoprotein cholesterol levels significantly differed between groups. In the low-albumin group, after adjusting for all confounding factors, the P value was 0.041 and the odds ratio (OR) was 4.608 [95% confidence interval (CI): 1.063, 19.063] compared with that in the normal-albumin group. Among male patients, considering all confounding factors, the OR was 12.936 (95% CI: 1.130, 148.125) and P = 0.040 in the low-albumin group compared with that for the normal-albumin group. No such relationship was found in female patients.

**Conclusion:**

A low albumin level is a risk factor for OP in the total population and in male patients but not in female patients. Therefore, albumin levels should be considered when controlling glucose levels in patients with T2DM.

## 1 Introduction

Osteoporosis (OP) is a systemic bone disease characterized by low bone mass, damage to bone microstructure, increased bone fragility, and susceptibility to fractures. Epidemiological data show that approximately 200 million people worldwide have OP, one-third of which are postmenopausal women (1). China’s Seventh National Population Census reveals a marked age-dependent increase in OP prevalence, with rates escalating from 19.2% in adults aged ≥50 years to 32.0% among those ≥65 years. Nationwide epidemiological estimates indicate approximately 90 million individuals currently affected by OP, 77.8% of whom (representing 70 million cases) are female (2).

OP is a silent disorder that is often asymptomatic. Several patients may not have been diagnosed with the disease until they experienced a fracture (3). Osteoporotic fractures pose a significant risk and are one of the main causes of disability and mortality in elderly patients. A meta-analysis suggests that the average mortality rate within 1 year after a hip fracture is 22% (4); approximately 50% of patients are disabled, and their quality of life significantly decreases (5). Moreover, medical and nursing care for OP and fractures can place a heavy burden on families and the society. The risk factors for OP include sex, smoking, alcohol consumption, and the use of glucocorticoids while other clinical factors, such as protein levels, may be related to OP. Previous studies have explored the relationship between albumin and OP; however, their conclusions have been inconsistent (6, 7).

Patients with type 2 diabetes mellitus (T2DM) demonstrate a 2-3 fold higher incidence of OP compared to non-diabetic populations, with diabetic OP now recognized as a distinct complication of chronic hyperglycemia (8). Paradoxically, while many patients adopt strict dietary regimens for glycemic control, prolonged nutritional restriction may inadvertently induce malnutrition-an established risk factor for BMD loss. In this study, we focused on patients with T2DM, to explore the relationship between OP and albumin, an indicator of nutrition.

## 2 Patients and methods

The registration number for this related study is ChiCTR2000029391 in ClinicalTrials.gov. This study protocol was approved by Hebei General Hospital Ethics Committee (ethical approval number: 2020–01) and complied with Declaration of Helsinki. All participants signed the informed consent prior to study.

### 2.1 Inclusion and exclusion criteria

This was an observational study. Participants were adult patients with T2DM hospitalized in the Department of Endocrinology between 2019 and 2020. OP was diagnosed according to bone mineral density (BMD) in line with World Health Organization guidelines and were based on dual-energy X-ray absorptiometer analysis: normal T-score: ≤–1; osteopenia T-score: <–2.5 to <–1; and OP T-score: ≤ –2.5 (9).

The exclusion criteria were as follows: (i) other types of diabetes, including type 1 diabetes, special type diabetes, and gestational diabetes; (ii) serious cardiovascular and cerebrovascular diseases or surgery within the past 3 months; (iii) severe urinary tract infections and lung infections; (iv) secondary OP; and (v) acute complications of diabetes, such as ketoacidosis, hyperglycemic hyperosmolar syndrome, and hypertonic syndrome.

### 2.2 Data collection

We screened patients based on inclusion and exclusion criteria and collected the following data from eligible patients: (i) general information: age, sex, smoking history, alcohol consumption history, family history of diabetes, use of insulin before administration, diabetes course, hypertension, and body mass index (BMI). (ii) Biochemical indicators: glycated hemoglobin (HbA1c), total protein, albumin, creatinine (Cr), urea nitrogen (BUN), total cholesterol (TC), triglyceride (TG), low-density lipoprotein-cholesterol (LDL-C), high-density lipoprotein-cholesterol (HDL-C), and very low-density lipoprotein-cholesterol (VLDL-C). (iii) Bone turnover markers (BTMs): 25-hydroxyvitamin D (25OHD), osteocalcin (OC), procollagen type 1 N-terminal propeptide (P1NP), β-C-terminal cross-linked telopeptide of type I collagen (β-CTX), and parathyroid hormone (PTH). Serum albumin levels were dichotomized using clinical thresholds: normal (≥30 g/L) versus low (<30 g/L).

### 2.3 Statistical analysis

SPSS 22.0 software was used for statistical analysis. For continuous data, normally distributed data were represented by mean ± standard deviation (SD), and an independent sample t-test was used for group comparison; non-normally distributed data were represented by median values (25th percentile, 75th percentile). The Wilcoxon Mann–Whitney test was used for group comparison. The Chi-square test was used for binary data, and the Wilcoxon Mann–Whitney test was used for graded data. For confounding factor screening, the P-value was limited to 0.1. Binary logistic regression was used to explore the relationship between albumin and OP. P < 0.05 was considered significant.

## 3 Results

### 3.1 Basic characteristics of participants

In total, 125 patients (68 men and 57 women) with T2DM were included in this study. The average age was 56.75 ± 11.32 years-old, the proportion of OP was 41.6%, the average level of albumin was 40.80 (38.75–42.74) g/L, and the average level of HbA1c was 8.60 (7.23–10.20) % as shown in **Table 1**.

**Table 1:** Clinical characteristics of patients with T2DM by OP.

	All participants (n=125)
Sex (M, %)	68 (54.4%)
Age (years)	56.75 ± 11.32
BMI (kg/m^2^)	25.38 ± 3.02
DM course (years)	
<1	20 (16.0%)
1-10	50 (40.0%)
≥10	55 (44.0%)
Family history of DM	44 (35.2%)
Hypertension	
No hypertension	69 (55.2%)
Grade 1	11 (8.8%)
Grade 2	16 (12.8%)
Grade 3	29 (23.2%)
Smoking history	36 (28.8%)
Alcohol consumption history	36 (28.8%)
Insulin Usage	51 (40.8%)
HbA1c (%)	8.60 (7.23, 10.20)
Lipid profiles	
TC (mmol/L)	4.60 (3.95, 5.23)
TG (mmol/L)	1.47 (1.06, 2.01)
HDL-C (mmol/L)	1.06 ± 0.23
LDL-C (mmol/L)	3.08 ± 0.80
VLDL-C (mmol/L)	0.52 ± 0.25
Total protein (g/L)	67.36 ± 6.28
Albumin (g/L)	40.80 (38.75, 42.74)
Low albumin (n, %)	47 (37.6%)
BUN (mmol/L)	4.85 (4.00, 5.90)
Cr (μmmol/L)	69.42 ± 13.88
Bone turnover biomarkers	
25OHD (ng/mL)	15.18 (12.42, 22.70)
OC (ng/mL)	14.60 (10.49, 18.30)
β-CTX (ng/mL)	0.41 (0.28, 0.62)
P1NP (ng/mL)	42.52 (33.10, 58.73)
PTH (pg/mL)	41.61 ± 14.38
OP (n, %)	52 (41.6%)

Data were expressed as number (%) or mean ± SD/median (P25, P75).

BMI, body mass index; BUN, blood urea nitrogen; Cr, creatinine; DM, diabetes mellitus; HbA1c, glycated hemoglobin; HDL-C, high-density lipoprotein cholesterol; LDL-C, low-density lipoprotein cholesterol; OC, osteocalcin; OP, osteoporosis; P1NP, procollagen type 1 N-terminal propeptide; PTH, parathyroid hormone; SD, standard deviation; T2DM, type 2 diabetes mellitus; TC, total cholesterol; TG, triglyceride; VLDL-C, very low-density lipoprotein cholesterol; 25OHD, 25-hydroxyvitamin D; β-CTX, β-C-terminal cross-linked telopeptide of type I collagen.

### 3.2 Comparisons between groups

Patients were divided into two groups according to the presence or absence of OP. TG levels in the OP group were significantly lower than those in the non-OP group (P < 0.05), and HDL-C levels were significantly higher in the OP group (P < 0.05) (**Table 2**).

**Table 2:** Clinical characteristics of patients with T2DM by OP.

	Non-OP group	OP group	P-value
n	73	52	
Sex (M, %)	42 (57.5%)	26 (50.0%)	0.404
Age (years)	56.78 ± 10.57	58.81 ± 11.34	0.307
BMI (kg/m^2^)	25.67 ± 3.01	25.17 ± 3.38	0.392
DM course (years)			0.862
<1	`13 (17.8%)	8 (15.4%)	
1-10	28 (38.4%)	21 (40.4%)	
≥10	32 (43.8%)	23 (44.2%)	
Family history of DM	29 (40.3%)	15 (29.4%)	0.215
Hypertension			0.899
No hypertension	40 (54.8%)	29 (55.8%)	
Grade 1	6 (8.2%)	5 (9.6%)	
Grade 2	10 (13.7%)	6 (11.5%)	
Grade 3	17 (23.3%)	12 (23.1%)	
Smoking history	25 (34.7%)	11 (21.6%)	0.114
Alcohol consumption history	23 (31.9%)	13 (25.5%)	0.438
Insulin Usage	31 (42.5%)	20 (38.5%)	0.653
HbA1c (%)	9.1 (7.9, 10.8)	8.9 (7.6, 10.3)	0.645
Lipid profiles			
TC (mmol/L)	4.72 (3.87, 5.28)	4.61 (3.90, 5.10)	0.950
TG (mmol/L)	1.71 (1.37, 2.44)	1.25 (0.91, 1.70)	0.003
HDL-C (mmol/L)	0.95 (0.84, 1.22)	1.07 (1.01, 1.24)	0.001
LDL-C (mmol/L)	3.01 ± 0.79	3.03 ± 0.82	0.935
VLDL-C (mmol/L)	0.48 (0.32, 0.72)	0.46 (0.33, 0.67)	0.069
Total protein (g/L)	67.15 ± 5.71	67.03 ± 6.31	0.912
Albumin (g/L)	40.92 ± 2.78	40.16 ± 2.94	0.145
Low albumin (n, %)	23 (31.5%)	24 (46.2%)	0.096
BUN (mmol/L)	5.32 ± 1.46	4.89 ± 1.35	0.093
Cr (μmmol/L)	74.90 (63.00, 83.00)	63.09 (58.49, 70.80)	0.055
Bone turnover markers			
25OHD (ng/mL)	15.11 (12.29, 23.18)	15.46 (12.42, 22.66)	0.837
OC (ng/mL)	14.03 ± 5.12	16.44 ± 5.92	0.207
β-CTX (ng/mL)	0.37 (0.25, 0.58)	0.43 (0.32, 0.65)	0.293
P1NP (ng/mL)	38.21 (29.75, 55.11)	47.57 (36.17, 61.29)	0.065
PTH (pg/mL)	42.29 ± 15.47	40.69 ± 15.74	0.601

Data were expressed as number (%) or mean ± SD/median (P25, P75).

BMI, body mass index; BUN, blood urea nitrogen; Cr, creatinine; DM, diabetes mellitus; HbA1c, glycated hemoglobin; HDL-C, high-density lipoprotein cholesterol; LDL-C, low-density lipoprotein cholesterol; OC, osteocalcin; OP, osteoporosis; P1NP, procollagen type 1 N-terminal propeptide; PTH, parathyroid hormone; SD, standard deviation; T2DM, type 2 diabetes mellitus; TC, total cholesterol; TG, triglyceride; VLDL-C, very low-density lipoprotein cholesterol; 25OHD, 25-hydroxyvitamin D; β-CTX, β-C-terminal cross-linked telopeptide of type I collagen.

In terms of stratification analysis by sex, among male patients, the TG and VLDL-C levels were significantly lower in the OP group than those in the non-OP group, whereas the HDL-C levels were significantly higher (P < 0.05) (**Table 3**). Among female patients, the PINP level was significantly higher in the OP group than that in the non-OP group (P < 0.05) (**Table 4**).

**Table 3:** Clinical characteristics of male patients with T2DM by OP.

	Non-OP group	OP group	P-value
n	42	26	
Age (years)	54.69 ± 10.61	56.5 ± 11.74	0.514
BMI (kg/m^2^)	26.01 ± 2.79	25.80 ± 3.61	0.789
DM course (years)			0.172
<1	5 (11.9%)	9 (34.6%)	
1-10	18 (42.9%)	7 (26.9%)	
≥10	19 (45.2%)	10 (38.5%)	
Family history of DM	15 (36.6%)	5 (20.0%)	0.155
Hypertension			0.477
No hypertension	19 (45.2%)	15 (57.7%)	
Grade 1	4 (9.5%)	2 (7.7%)	
Grade 2	8 (19.0%)	2 (7.7%)	
Grade 3	11 (26.2%)	7 (26.9%)	
Alcohol consumption history	25 (61.0%)	11 (44.0%)	0.179
Drinking history	22 (53.7%)	13 (52.0%)	0.896
Insulin Usage	19 (45.2%)	9 (34.6%)	0.387
HbA1c (%)	8.82 ± 1.93	8.63 ± 2.82	0.735
Lipid profiles			
TC (mmol/L)	4.50 ± 1.01	4.61 ± 1.08	0.662
TG (mmol/L)	1.88 (1.44, 2.38)	1.17 (0.90, 1.69)	0.009
HDL-C (mmol/L)	0.88 (0.81, 1.24)	1.07 (1.00, 1.25)	0.008
LDL-C (mmol/L)	2.93 ± 0.74	3.05 ± 0.82	0.545
VLDL-C (mmol/L)	0.45 (0.31, 0.64)	0.42 (0.33, 0.52)	0.030
Total protein (g/L)	67.20 ± 6.06	65.82 ± 5.13	0.339
Albumin (g/L)	41.12 ± 3.05	40.50 ± 2.82	0.408
Low albumin (n, %)	12 (28.6%)	12 (46.2%)	0.140
BUN (mmol/L)	5.39 ± 1.52	5.04 ± 1.22	0.332
Cr (μmmol/L)	73.80 (65.73, 81.83)	63.32 (59.60, 70.55)	0.391
Bone turnover markers			
25OHD (ng/mL)	17.37 ± 5.42	16.90 ± 6.72	0.772
OC (ng/mL)	13.97 ± 4.84	16.21 ± 5.72	0.112
β-CTX (ng/mL)	0.35 (0.25, 0.51)	0.41 (0.23, 0.64)	0.962
P1NP (ng/mL)	44.01 (29.75, 54.48)	42.01 (35.89, 52.13)	0.667
PTH (pg/mL)	42.13 ± 16.04	36.39 ± 12.26	0.149

Data were expressed as number (%) or mean ± SD/median (P25, P75).

BMI, body mass index; BUN, blood urea nitrogen; Cr, creatinine; DM, diabetes mellitus; HbA1c, glycated hemoglobin; HDL-C, high-density lipoprotein cholesterol; LDL-C, low-density lipoprotein cholesterol; OC, osteocalcin; OP, osteoporosis; P1NP, procollagen type 1 N-terminal propeptide; PTH, parathyroid hormone; SD, standard deviation; T2DM, type 2 diabetes mellitus; TC, total cholesterol; TG, triglyceride; VLDL-C, very low-density lipoprotein cholesterol; 25OHD, 25-hydroxyvitamin D; β-CTX, β-C-terminal cross-linked telopeptide of type I collagen.

**Table 4:** Clinical characteristics of female patients with T2DM by OP.

	Non-OP group	OP group	P-value
n			
Age (years)	59.61 ± 10.00	61.12 ± 10.64	0.585
BMI (kg/m^2^)	24.65 (23.15, 26.61)	25.66 (22.79, 26.13)	0.396
DM course (years)			0.777
<1	2 (6.5%)	4 (15.4%)	
1-10	15 (48.4%)	10 (38.5%)	
≥10	14 (45.2%)	12 (46.2%)	
Family history of DM	14 (45.2%)	10 (38.5%)	0.610
Hypertension			0.398
No hypertension	21 (67.7%)	14 (53.8%)	
Grade 1	2 (6.5%)	3 (11.5%)	
Grade 2	2 (6.5%)	4 (15.4%)	
Grade 3	6 (19.4%)	5 (19.2%)	
Insulin Usage	12 (38.7%)	11 (42.3%)	0.783
HbA1c (%)	9.04 ± 1.95	9.30 ± 2.10	0.630
Lipid profiles			
TC (mmol/L)	4.52 (3.87, 5.74)	4.74 (4.04, 5.10)	0.898
TG (mmol/L)	1.70 (1.13, 2.82)	1.38 (0.91, 2.24)	0.265
HDL-C (mmol/L)	1.02 ± 0.22	1.14 ± 0.24	0.058
LDL-C (mmol/L)	3.17 (2.67, 3.80)	3.01 (2.66, 3.20)	0.631
VLDL-C (mmol/L)	0.58 ± 0.27	0.55 ± 0.32	0.723
Total protein (g/L)	67.09 ± 5.30	68.24 ± 7.20	0.502
Albumin (g/L)	40.65 ± 2.41	39.82 ± 3.07	0.256
Low albumin (n, %)	11 (35.5%)	12 (46.2%)	0.413
BUN (mmol/L)	5.22 ± 1.38	4.73 ± 1.47	0.200
Cr (μmmol/L)	68.72 ± 14.50	64.33 ± 11.60	0.221
Bone turnover markers			
25OHD (ng/mL)	13.63 (11.98, 18.97)	16.74 (12.96, 22.47)	0.983
OC (ng/mL)	14.11 ± 5.58	16.68 ± 6.25	0.140
β-CTX (ng/mL)	0.44 ± 0.24	0.52 ± 0.21	0.305
P1NP (ng/mL)	37.64 (30.26, 55.34)	54.82 (46.76, 65.42)	0.032
PTH (pg/mL)	42.52 ± 14.95	45.19 ± 17.90	0.676

Data were expressed as number (%) or mean ± SD/median (P25, P75).

BMI, body mass index; BUN, blood urea nitrogen; Cr, creatinine; DM, diabetes mellitus; HbA1c, glycated hemoglobin; HDL-C, high-density lipoprotein cholesterol; LDL-C, low-density lipoprotein cholesterol; OC, osteocalcin; OP, osteoporosis; P1NP, procollagen type 1 N-terminal propeptide; PTH, parathyroid hormone; SD, standard deviation; T2DM, type 2 diabetes mellitus; TC, total cholesterol; TG, triglyceride; VLDL-C, very low-density lipoprotein cholesterol; 25OHD, 25-hydroxyvitamin D; β-CTX, β-C-terminal cross-linked telopeptide of type I collagen.

In our cohort of 125 patients, 37.6% (47/125) exhibited hypoalbuminemia. Initial comparisons showed no significant difference in continuous albumin levels between OP and non-OP groups (40.16 ± 2.94 vs 40.92 ± 2.78 g/L, p=0.145). However, categorical analysis revealed a non-significant trend toward higher prevalence of hypoalbuminemia in OP patients (46.2% [24/52] vs 31.5% [23/73], p=0.096). Notably, this pattern remained consistent across sex-stratified analyses, with neither males nor females showing significant albumin-OP associations in either continuous or categorical comparisons (all p>0.10).

### 3.3 Regressive analysis

With a P-value of 0.1 as the threshold, significant differences were present in the levels of TG, HDL-C, VDL-C, BUN, Cr, and PINP between the groups. Age, BMI, smoking history, alcohol consumption history, total protein, and all BTMs were considered confounding factors in the clinical analysis.

Using binary regression analysis in Model 1 (without adjusting for confounding factors), the P-value was 0.067. In Model 2, after adjusting for confounding factors (age, sex, smoking history, alcohol consumption history, BMI, and total protein level), the P-value was 0.096. In Model 3, after adjusting for all confounding factors, the P-value was 0.041 and the OR was 4.608 (95% CI: 1.063,19.063), indicating that the risk of OP in the low-albumin group was -3.608-fold higher than that in the normal-albumin group (**Table 5**).

**Table 5:** Logistic regression model of albumin categories for OP in patients with T2DM adjusting for covariates.

	Normal albumin	Low albumin	P for trend
Model 1	1.00 (reference)	1.863 (0.893, 3.888)	0.097
Model 2	1.00 (reference)	2.229 (0.868, 5.728)	0.096
Model 3	1.00 (reference)	4.608 (1.063, 19.063)	0.041

Data were expressed as OR (95% CI).

Model 1: crude model.

Model 2: adjusted for age, sex, smoking history and alcohol consumption history, BMI and total protein.

Model 3: adjusted for age, sex, smoking history, alcohol consumption history, BMI, total protein, TG, HDL-C, VLDL-C, BUN, Cr, 25OHD, OC, β-CTX, P1NP and PTH.

BMI, body mass index; BUN, blood urea nitrogen; CI, confidence interval; Cr, creatinine; HDL-C, high-density lipoprotein cholesterol; T2DM, type 2 diabetes mellitus; TG, triglyceride; VLDL-C, very low-density lipoprotein cholesterol; OC, osteocalcin; OP, osteoporosis; OR, odds ratio; P1NP, procollagen type 1 N-terminal propeptide; PTH, parathyroid hormone; 25OHD, 25-hydroxyvitamin D; β-CTX, β-C-terminal cross-linked telopeptide of type I collagen.

Among male patients, confounding factors included age, smoking history, alcohol consumption history, BMI, total protein, TG, HDL-C, VLDL-C, BTMs. P values were 0.144 in Model 1 (without adjusting for confounding factors) and 0.339 in Model 2 (adjusted for some confounding factors), with no statistical significance. In Model 3, after adjusting for all confounding factors, the OR was 12.936 (95% CI: 1.130, 148.125) and P = 0.040, suggesting that in male patients with T2DM, the risk of OP in the low-albumin group was 11.936-fold higher than that in the normal-albumin group (**Table 6**).

**Table 6:** Logistic regression model of albumin categories for OP in male patients with T2DM adjusting for covariates.

	Normal albumin	Low Albumin	P for trend
Model 1	1.00 (reference)	2.143 (0.772, 5.949)	0.144
Model 2	1.00 (reference)	1.893 (0.512, 7.002)	0.339
Model 3	1.00 (reference)	12.936 (1.130, 148.125)	0.040

Data were expressed as OR (95% CI).

Model 1: crude model.

Model 2: adjusted for age, smoking history and alcohol consumption history BMI and total protein.

Model 3: adjusted for age, smoking history, alcohol consumption history, BMI, total protein, TG, HDL-C, VLDL-C, 25OHD, OC, β-CTX, P1NP and PTH.

BMI, body mass index; CI, confidence interval; HDL-C, high-density lipoprotein cholesterol; OC, osteocalcin; OP, osteoporosis; OR, odds ratio; P1NP, procollagen type 1 N-terminal propeptide; PTH, parathyroid hormone; T2DM, type 2 diabetes mellitus; TG, triglyceride; VLDL-C, very low-density lipoprotein cholesterol; 25OHD, 25-hydroxyvitamin D; β-CTX, β-C-terminal cross-linked telopeptide of type I collagen.

Among female patients, confounding factors included age, BMI, total protein, HDL-C, BTMs. Regression analysis found that no significant difference was present in the risk of OP between the low- and normal-albumin groups both without adjustment for confounding factors (Model 1) and with adjustment for confounding factors (Models 2 and 3) (**Table 7**).

**Table 7:** Logistic regression model of albumin categories for OP in male patients with female T2DM adjusting for covariates.

	Normal albumin	Low Albumin	P for trend
Model 1	1.00 (reference)	1.558 (0.537, 4.524)	0.415
Model 2	1.00 (reference)	4.122 (0.867, 19.607)	0.075
Model 3	1.00 (reference)	7.689 (0.634, 93.277)	0.109

Data were expressed as OR (95% CI).

Model 1: crude model.

Model 2: adjusted for age, BMI, total protein.

Model 3: adjusted for age, BMI, total protein, HDL-C, TG, 25OHD, OC, β-CTX, P1NP and PTH.

BMI, body mass index; CI, confidence interval; HDL-C, high-density lipoprotein cholesterol; OC, osteocalcin; OP, osteoporosis; OR, odds ratio; P1NP, procollagen type 1 N-terminal propeptide; PTH, parathyroid hormone; T2DM, type 2 diabetes mellitus; TG, triglyceride; 25OHD, 25-hydroxyvitamin D; β-CTX, β-C-terminal cross-linked telopeptide of type I collagen.

## 4 Discussion

Albumin levels <35 g/L are considered to be indicative of hypoalbuminemia, whereas those between 35 and 40 g/L are mildly reduced, and >40 g/L are normal. In our study, albumin levels were not classified into the above three categories because only three patients with albumin levels <35 mg/dL were included in our data. Consequently, we divided the patients into low-albumin and control groups. No obvious hypoalbuminemia was observed in patients were hospitalized in our department and these may have a relatively mild disease status and acceptable nutritional status.

Albumin is the most abundant protein in blood and has various physiological functions, including osmotic pressure, molecular transport, anti-inflammatory, antioxidant, and endothelial stability. Hypoalbuminemia is associated with various metabolic disorders such as malignant tumors, nephrotic syndrome, and inflammation-malnutrition syndrome (10). Studies have shown that hypoalbuminemia may be associated with BMD and OP. Farsad’s study included 15,539 subjects from the National Health and Nutrition Examination Survey; after adjusting for confounding factors, the incidence rate of osteoporotic fractures in the femoral neck and lumbar spine in the hypoproteinemia group was 5.37- and 4.59-fold higher than that in the normal-albumin group. In another study, hypoalbuminemia was found to be closely associated with OP in a dose- and duration-dependent manner. Lower albumin levels and longer durations were associated with a greater risk of OP (11). However, previous studies have shown to have opposing results; for example, Bernardo et al. found that, after adjusting for confounding factors such as age, no significant correlation was present between the albumin level and BMD (12). In addition, the serum albumin level does not play a significant role in the pathogenesis of BMD reduction in healthy postmenopausal women (13).

Several mechanisms may underlie the relationship between low albumin levels, BMD, and OP. First, hypoalbuminemia can directly activate osteoclasts and inhibit osteoblasts from passing through the nuclear factor-κB (14). Second, chronic low albumin levels indicate prolonged protein deficiency and malnutrition, which can weaken bone formation by reducing collagen production and IGF-1 availability – key factors for bone strength (15). Third, in T2DM, ongoing inflammation (with high IL-6/TNF-α levels) further damages bones by increasing osteoclast activity. Low albumin worsens this process because albumin normally helps neutralize inflammatory molecules that drive bone loss (16).

The target population of our study was patients with T2DM who suffered damage to multiple systems, including the cardiovascular system, eyes, and kidneys, and also the impact on bone metabolism. Patients with diabetes must balance their diet to control blood glucose; however, their nutritional status should be assessed. In patients with T2DM, diabetes accelerates the loss of muscle strength, mass, and serum albumin, drawing attention to the protein and energy balance (17). Protein supplementation can increase muscle strength and body stability (18) and increasing food protein intake increases insulin growth factor-1 levels (enhancing bone strength) (19). Among patients with T2DM, the Geriatric Nutritional Risk Index (calculated using factors such as albumin and body weight) is positively correlated with bone density and negatively correlated with the incidence rate of OP (20).

When conducting the regression analysis, we included confounding factors such as age, smoking history, alcohol consumption history, BMI, total protein, and BTMs. In addition, we limited the value to within 0.1 for statistically significance when comparing the groups. The specific reasons for this are as follows. OP is an age-related disease. The incidence rate of OP increases with age. In addition, smoking and alcohol consumption can increase the risk of developing OP. Our previous study found that BMI and TG are closely related to OP and that these are independent protective factors against OP in patients with T2DM (21). Furthermore, we also included BTMs, including bone formation markers (25OHD, OC, and P1NP) and bone destruction markers (β- CTX and PTH) (22).

For sex differences, we conducted a stratified analysis because the incidence rate in postmenopausal women is relatively high. This study found that in the general population, after adjusting for confounding factors, low albumin levels increased the risk of OP by 3.608–fold, and in male patients with type T2DM, the low-albumin group increased the risk of OP by 11.936-fold. However, no significant difference occurred among the female patients. Consequently, these patients may be better analyzed as subgroups because they included both premenopausal and postmenopausal women.

This study has some limitations. First, this is an observational study that explored the relationship between the albumin level and OP in T2DM, although we could not obtain a causal relationship between the two, which may require more rigorous basic and clinical research. Second, the sample size was relatively small, and studies with larger sample sizes are more convincing. Third, a degree of bias may be present in the smoking and alcohol consumption history records. Women may be less willing to be admitted to hospital. Fourth, some factors affecting bone metabolism were not considered in our study. For instance, specific antidiabetic agents—including SGLT-2 inhibitors and thiazolidinediones—merit special consideration given their well-documented associations with higher risk of OP. Additionally, prolonged diabetes duration and postmenopausal estrogen deficiency are established contributors to OP. In future studies, when developing research protocols, we need to consider all the possible influencing factors to facilitate subsequent statistical analysis. Last, our study enrolled hospitalized patients with T2DM, many of whom were admitted specifically for hyperglycemia. Consequently, these patients exhibited relatively high HbA1c levels, which may have introduced some degree of bias.

## 5 Conclusion

In conclusion, in male patients with T2DM, low albumin levels are a risk factor for OP, and this relationship was not found in female patients. Therefore, attention should be paid to albumin levels and increase them appropriately when controlling glucose levels in patients with T2DM.

## Data Availability

The original contributions presented in the study are included in the article/supplementary material. Further inquiries can be directed to the corresponding authors.
